# Flagella-Mediated Adhesion and Extracellular DNA Release Contribute to Biofilm Formation and Stress Tolerance of *Campylobacter jejuni*

**DOI:** 10.1371/journal.pone.0106063

**Published:** 2014-08-28

**Authors:** Sarah L. Svensson, Mark Pryjma, Erin C. Gaynor

**Affiliations:** Department of Microbiology and Immunology, University of British Columbia, Vancouver, British Columbia, Canada; East Carolina University School of Medicine, United States of America

## Abstract

*Campylobacter jejuni* is a leading cause of foodbourne gastroenteritis, despite fragile behaviour under standard laboratory conditions. In the environment, *C. jejuni* may survive within biofilms, which can impart resident bacteria with enhanced stress tolerance compared to their planktonic counterparts. While *C. jejuni* forms biofilms *in vitro* and in the wild, it had not been confirmed that this lifestyle confers stress tolerance. Moreover, little is understood about molecular mechanisms of biofilm formation in this pathogen. We previously found that a Δ*cprS* mutant, which carries a deletion in the sensor kinase of the CprRS two-component system, forms enhanced biofilms. Biofilms were also enhanced by the bile salt deoxycholate and contained extracellular DNA. Through more in-depth analysis of Δ*cprS* and WT under conditions that promote or inhibit biofilms, we sought to further define this lifestyle for *C. jejuni*. Epistasis experiments with Δ*cprS* and flagellar mutations (Δ*flhA*, Δ*pflA*) suggested that initiation is mediated by flagellum-mediated adherence, a process which was kinetically enhanced by motility. Lysis was also observed, especially under biofilm-enhancing conditions. Microscopy suggested adherence was followed by release of eDNA, which was required for biofilm maturation. Importantly, inhibiting biofilm formation by removal of eDNA with DNase decreased stress tolerance. This work suggests the biofilm lifestyle provides *C. jejuni* with resilience that has not been apparent from observation of planktonic bacteria during routine laboratory culture, and provides a framework for subsequent molecular studies of *C. jejuni* biofilms.

## Introduction

*Campylobacter jejuni* is a prevalent food- and waterborne gastrointestinal pathogen. Infection commonly presents as an acute gastroenteritis, marked by fever, stomach cramps, and diarrhea. Although illness is usually self-limiting, the high incidence of infection, together with the significant subset of cases that go on to manifest as serious autoimmune sequelae (such as Guillain-Barré syndrome), contributes to the significant burden of *C. jejuni* infection. Preventative strategies that limit *C. jejuni* exposure and infection may thus greatly reduce its impact.

Mechanisms by which *C. jejuni* causes disease are relatively enigmatic, suggesting it may use unique strategies compared to more extensively characterized enteric pathogens. In fact, many factors identified as critical to pathogenesis include those related to survival of stress and basic biology of the organism, including the stringent response, motility, and surface carbohydrates [1], [2]. In the gastrointestinal tract of commensal or susceptible animals, *C. jejuni* tolerates insults such as bile salts. Moreover, *C. jejuni* is zoonotic, with sporadic cases associated with consumption of undercooked poultry and outbreaks arising from contaminated water, and thus survives transmission environments characterized by a range of nutrient availabilities, temperatures, oxygen tensions, and osmolarities.

Understanding how *C. jejuni* survives in such environments may help direct strategies to limit its impact. However, inspection of the genome of numerous strains suggests lacks many classical stress tolerance factors, including the RpoS stationary phase sigma factor [3]. *C. jejuni* is also relatively fragile and fastidious in the laboratory, with specific atmospheric and nutrient requirements for growth, bringing into question how it can adapt to environments both inside and outside the host. *C. jejuni* may serve as a model for understanding how pathogens with limited regulatory repertoires adapt to pathogenesis-related environments.

The paradox of *C. jejuni*’s success may be explained by a tendency to persist in distinct lifestyles in natural environments. Phenotypes displayed during logarithmic growth in rich broth may not be representative of phenotypes expressed in nature. In fact, most bacteria exist naturally in sessile biofilms: adhered communities of microorganisms encased in a polymeric extracellular matrix. Formation of a biofilm proceeds in a set of distinct steps (adherence, microcolony formation, matrix release, dispersal) that have been proposed to represent ‘microbial development’ [4]. The mechanisms and factors that underlie each step appear distinct for each bacterial species. For example, attachment may be mediated by flagella, pili, carbohydrates, or protein adhesins, and the biofilm matrix can be a unique mixture of hydrated extracellular polymeric substances, such as carbohydrates, proteins, and lipids [5]. The significant contribution of extracellular DNA (eDNA) to biofilm structure and function, including structural integrity, recombination, and antibiotic resistance, has been also recently become apparent [6], [7]. Autolysis can underlie either biofilm formation or dispersal, and can release eDNA [6], [8], [9].

Biofilms have been proposed to contribute to survival of *C. jejuni* in the food chain, from farm to fork [10]. However, the contribution of biofilms to *C. jejuni* resilience is unclear. Observation of *C. jejuni* in the wild, such as within biofilms in aquatic environments suggests that biofilm residents display several phenotypic differences from their planktonic counterparts, including enhanced stress tolerance. There is some evidence that *C. jejuni* cells residing within biofilms in aquatic environments survive better than their planktonic counterparts [11]–[15]. Strains residing within chicken house drinking water biofilms have also been found to colonize broiler flocks [16], and *C. jejuni* forms biofilms *in vitro* under conditions that may mimic environments encountered during pathogenesis. Biofilm formation is affected by nutrients, temperature, oxygen tension, and osmolarity [17], [18] and notably, enhanced in the presence of the bile salt sodium deoxycholate (DOC) [19]. Although biofilm-enhanced mutants have been identified, including Δ*ppk1*, Δ*ppk2*, Δ*spoT*, Δ*peb4*, Δ*kpsM*, and Δ*waaF* [20]–[24], demonstration of increased tolerance by such strains is hampered by the pleiotropic effects of such mutations. Molecular factors that mediate *C. jejuni* biofilm formation are also poorly understood. Flagella are required [17], [25], [26] and may mediate adhesion - both bacteria-bacteria and biofilm-host cell [27]–[29]. Whether motility or the flagellar structure itself is important, and at which stage each is required, is unclear. Moreover, the biofilm matrix of such a “sugary” bug remains surprisingly ill-defined: while carbohydrate changes correlate with biofilm formation [22], [23], a carbohydrate component has not been definitively identified. Instead, extracellular DNA has been observed in *C. jejuni* biofilms [19], [30].

We previously identified a two-component regulatory system, CprRS (*Campylobacter* planktonic growth regulation), which may control phenomena central to biofilm formation [19]. A Δ*cprS* sensor kinase mutant forms markedly enhanced biofilms compared to the parental strain, but has no obvious differences in carbohydrate production. Here, we extend characterization of this strain to provide insight into mechanisms of *C. jejuni* biofilm formation through exploration of the temporal development of *C. jejuni* biofilms using confocal microscopy. We show that flagella are required for initial attachment of biofilms, and that eDNA is dispensable for this step. We have also find evidence of a lytic process that correlates with biofilm maturation and releases eDNA. Finally, we provide evidence that inhibition of biofilm formation affects the fitness of *C. jejuni*, and that biofilm formation may contribute to long-term survival of *C. jejuni* populations by contributing to genetic diversity.

## Materials and Methods

### Bacterial strains and routine culture conditions

*C. jejuni* strain 81–176, a highly invasive isolate from a raw milk outbreak [31], was used as the WT parental strain. All other strains are listed in **Table 1**. Targeted deletion mutants, such as Δ*cprS*, Δ*flhA*, and Δ*flgR,* have been described previously [19], [32]. The Δ*pflA* mutant was isolated from a transposon mutant screen using the Mariner system developed for *C. jejuni* [33]. The double Δ*cprS* Δ*flhA* mutant was constructed by naturally transforming genomic DNA from Δ*flhA* into Δ*cprS* and selecting for Kan^R^ Cm^R^ colonies. *C. jejuni* strains were routinely cultured in Müller-Hinton (MH) broth (Oxoid, Hampshire, England) or on MH agar (1.7%) plates at 37°C under microaerobic conditions (6% O_2_, 12% CO_2_) in a Sanyo tri-gas incubator (plates and standing liquid cultures/biofilms) or generated using the Oxoid CampyGen system (shaking broth cultures). All media were supplemented with 10 µg mL^−1^ vancomycin and 5 µg mL^−1^ trimethoprim (Sigma, Oakville, ON). Where appropriate, antibiotics kanamycin (Kan), chloramphenicol (Cm), and streptomycin (Str) were added to a final concentration of 40 µg mL^−1^, 15 µg mL^−1^, and 100 µg mL^−1^, respectively.

**Table 1:** Strains used in this study.

Strain	Genotype	Relevant characteristics	Source
*C. jejuni* strains			
81–176	Wild type (WT)		Korlath *et al.* 1985
DRH461	Δ*astA*	Str^R^	
Δ*cprS*	Δ*cprS*::*aph3*	biofilm-enhanced, Kan^R^	Svensson *et al.* 2009
Δ*flhA*	*flhA*::*cat*-*rpsL*	aflagellate, non-motile, Cm^R^	Hendrixson and DiRita 2003
Δ*cprS* Δ*flhA*	Δ*cprS*::*aph3 flhA*::*cat*-*rpsL*	aflagellate, non-motile, Kan^R^ Cm^R^	this study
Δ*pflA*	*pflA*::*solo*	paralyzed flagella, non-motile, Kan^R^	laboratory collection
Δ*flgR*	*flgR*::*kan*-*rpsL*	aflagellate, non-motile, Kan^R^	Hendrixson and DiRita 2003
Plasmids			
P*_atpF_*-*gfp* pRY112	GFP under control of the *atpF*’ promoter in pRY112	Cm^R^	Apel *et al.* 2012

### Crystal violet biofilm assay

Biofilm formation was assessed as described previously [19], [20], [22], [23], [34], [35]. Values shown are the mean A_570_ +/− standard error of three biofilm cultures for each strain/condition. To determine statistical significance, an unpaired *t*-test was performed with significance set at p<0.05. Where indicated, DNase I (Invitrogen) was added to a final concentration of 90 U mL^−1^, and sodium deoxycholate (DOC, Sigma) was included at 0.05%. In some experiments, 50 µL of WT culture supernatant, isolated as described below, was added to each mL of fresh MH broth. Genomic DNA was isolated from WT *C. jejuni* grown for 24 h on MH agar using the Qiagen genomic tip 100/G kit and was added, where indicated, at a concentration of 500 ng µL^−1^.

### Standing culture growth

Standard biofilm cultures of each strain (WT, Δ*cprS*, Δ*flhA*, and Δ*cprS* Δ*flhA*) were inoculated to an OD_600_ of 0.002 in either MH broth only, MH/DOC (0.05%), MH/DNase (90 U mL^−1^), or MH/DOC/DNase. Following 2 days of growth under microaerobic conditions, tubes were either stained with crystal violet to assess biofilm formation, or vortexed for one min., followed by measuring OD_600_.

### Detection of bacterial cell lysis

Lysis was assessed by SDS-PAGE and Western blot analysis of culture supernatants. Following growth in shaking broth culture (10 mL) for 24 h, a 1 mL sample of culture was harvested for analysis of total cellular protein expression. Cells from the rest of the culture were removed by centrifugation at 10,000×g for 5 min. and discarded. Any cells remaining in this clarified supernatant were removed by filtration through a 0.22 µM filter. Supernatants were then concentrated approximately 10-fold from 2.5 mL to 250 µL using 3 kDa cutoff Amicon Ultra centrifugal filter units (Millipore, Billerica, MA) by centrifugation for 60 min. at 4,000 x g. Samples were then analyzed by SDS-PAGE/Western blotting with an anti-CosR antibody (a gift from Dr. Stu Thompson).

### Quantification of eDNA

The amount of DNA present in culture supernatants was measured by QPCR. Supernatants, prepared as above, were used as templates for qPCR using primers *cprR-*QPCRFWD/REV (5′-GACCTTTCTTTGCCAGGGCTTGAT and 5′-GGTAGGTAATCATCTGCTCCAAGCTC, respectively). QPCR was performed in triplicate on equal volumes (0.5 µL) of supernatant as template using IQ SYBR Green Supermix and the MyIQ Real-time PCR Detection System (Biorad, Mississauga, ON) according to the manufacturer’s specifications.

### Confocal microscopy of biofilms

For confocal microscopy, a plasmid encoding green fluorescent protein (GFP) expressed from the *atpF*’ promoter [36] was introduced into strains by natural transformation. Biofilm cultures were set up as in above, except a glass coverslip was included standing upright in each tube. MH broth was supplemented with Cm for plasmid selection. Following 12, 24, or 36 h, culture medium was removed and biofilms were fixed by the addition of 4% paraformaldehyde in PBS, pH 7.4 for 15 min. Fixing solution was removed and replaced with PBS, and coverslips were stored at 4°C. Samples were mounted using Prolong Gold Antifade with DAPI (4′,6-diamino-2-phenylindol; Invitrogen), and imaging was performed with an Olympus Fluoview FV1000 laser scanning confocal microscope with FV10-ASW 2.0 Viewer software to adjust images.

### Measurement of recombination

Transfer of resistance markers between strains was measured in mixed-strain shaking broth cultures. Each strain was marked (on the chromosome) with a different antibiotic resistance marker and recombination was determined by measuring appearance of doubly resistant recombinant clones. Briefly, WT (marked with Str^R^) was grown in mixed culture (1∶1) with either an isogenic WT strain (marked with Kan^R^; insertion into an rRNA spacer via pRRK) or the Δ*cprS* hyper-biofilm mutant (marked with Kan^R^; allelic replacement of the *cprS* locus). Cultures were grown in either MH alone or MH/DOC (0.05%). Cells were removed A) immediately following inoculation and B) following 8 h growth and plated on MH agar with the appropriate antibiotics (Kan, Cm, and/or Str) for determination of colony-forming units (CFUs).

## Results

### Strains grown under conditions that promote biofilms show enhanced and accelerated appearance of eDNA

**Figure 1:**
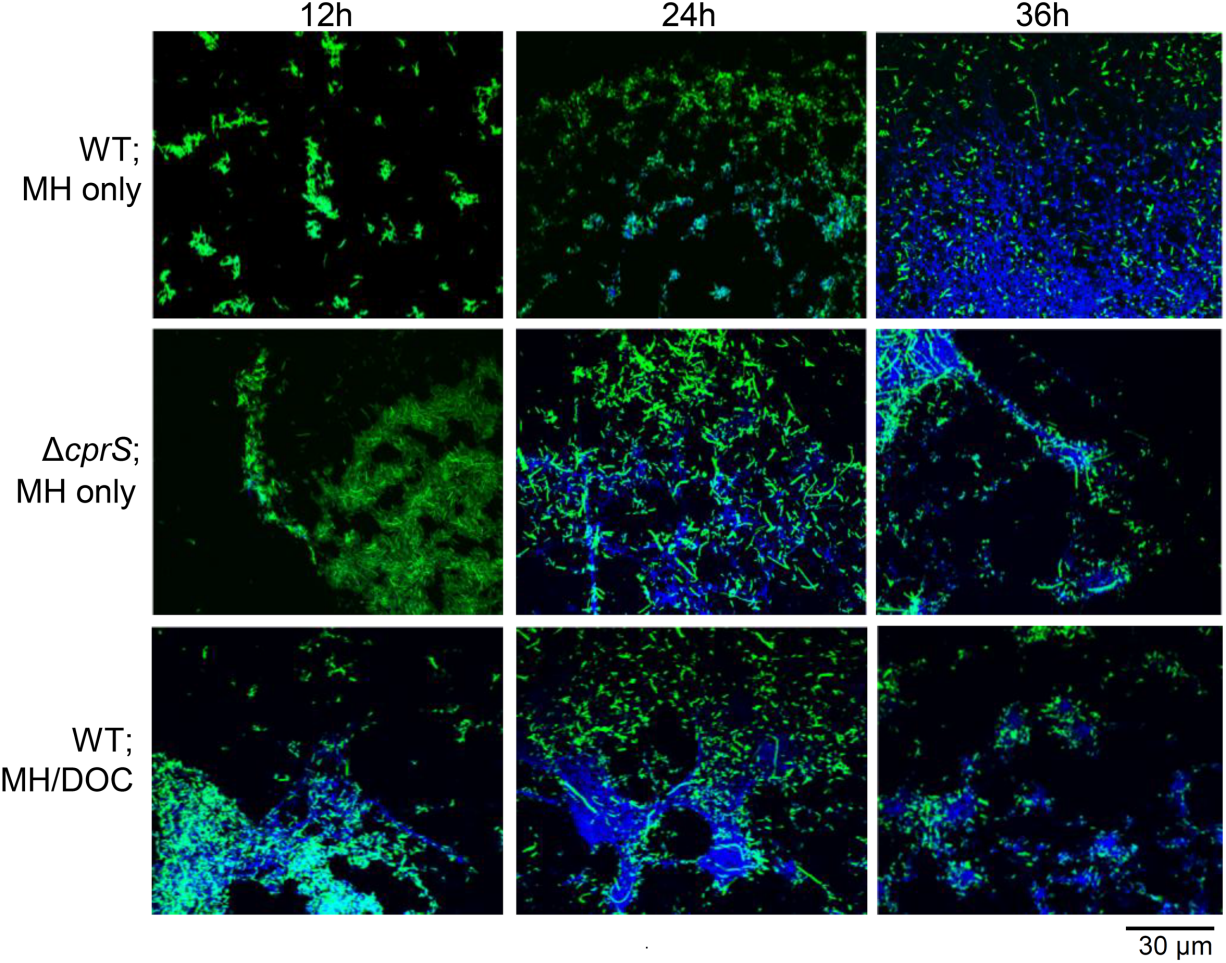
DNA appears in WT biofilms following attachment and is more pronounced under conditions that promote biofilm formation. Biofilms of WT or Δ*cprS* were grown on glass coverslips in MH broth alone or MH/DOC (0.05%). At indicated times post-inoculation, coverslips were fixed, stained with DAPI, and visualized by confocal microscopy. Green: GFP-expressing bacteria; Blue: DAPI-stained DNA.

We previously noted extracellular DNA (eDNA) in *C. jejuni* biofilms, and thatthe amount of eDNA appeared to be qualitatively increased in strains forming enhanced biofilms, such as Δ*cprS* and WT in MH/deoxycholate (DOC; a bile salt) [19]. Furthermore, Production of a specific surface polysaccharide does not appear to correlate with *C. jejuni* biofilm formation, unlike in other bacteria. We thus hypothesized that eDNA could instead be a marker for *C. jejuni* biofilm formation. To this end, the temporal relationship between biofilm formation and appearance of eDNA was examined by confocal microscopy observation of biofilms in a time course experiment (**Fig. 1**). GFP-expressing bacteria were inoculated into MH broth standing cultures (WT and Δ*cprS*). WT grown in MH/DOC was also included for comparison. At different time points post-inoculation (12, 24, 36 h), biofilms were fixed, stained with DAPI, and samples were observed by confocal microscopy.

For WT under routine laboratory conditions (top panels, ‘WT; MH alone’), green bacteria first adhered to the coverslip in small microcolonies (12 h). This was followed, at 24 h, by the appearance of blue DNA. DNA, likely extracellular due to its mucoid appearance, was more prevalent in regions closer to the interior of the biofilm. As time progressed to 36 h, the amount of eDNA and the apparent thickness of the biofilm increased further. This suggested that DNA not only was a quantitative marker for biofilm formation, but was also a temporal marker as it was not present in appreciable amounts upon biofilm initiation.

We next compared kinetics for a strain and condition previously shown to enhance biofilm formation (middle and bottom panels, ‘Δ*cprS’* and ‘WT in MH/DOC’). A similar temporal relationship between initiation and DNA appearance was seen for both biofilm-enhanced cultures. However, the process appeared both accelerated and markedly enhanced. For example, at 12 h, we generally observed that Δ*cprS* microcolonies were markedly larger than those observed for WT. The heterogeneous nature of the biofilms formed on coverslips precluded quantification of thickness for comparison. However, Δ*cprS* and WT in MH/DOC generally appeared thicker, compared to WT in MH alone, at this time point. Furthermore, foci of blue DNA were already visible at 12 h in some regions of Δ*cprS* biofilms. By 36 h, even larger strands of eDNA were observed. WT in MH/DOC appeared more accelerated and enhanced than Δ*cprS*. For example, at 12 h, had significant amounts of blue eDNA present. For both Δ*cprS* and WT in MH/DOC at 36 h, less overall coverage of both bacteria and DNA on the coverslip likely reflects some sloughing off of very thick biofilms. Together, observations of both WT and enhanced *C. jejuni* biofilms suggest that the appearance of eDNA coincides with maturation and may be a temporal, and potentially quantitative, marker for biofilm formation in *C. jejuni*.

### Conditions that increase extracellular DNA also correlate with bacterial lysis

**Figure 2:**
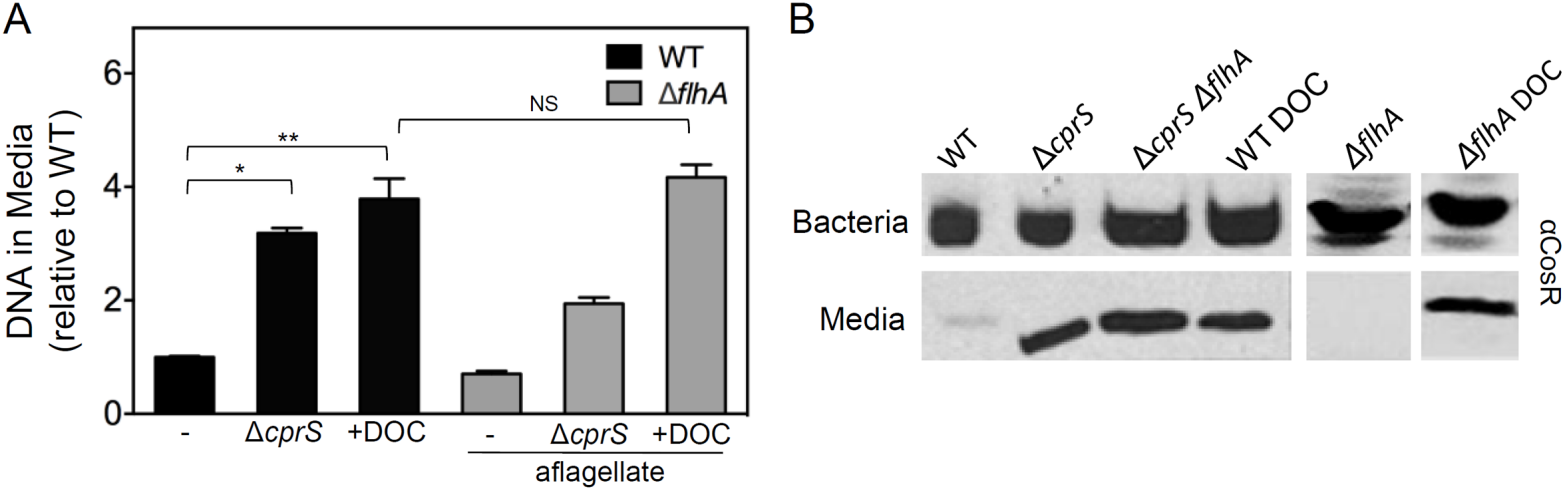
Increased extracellular DNA and lysis occur in biofilm-enhanced cultures. **A**) Cell-free supernatants contain more DNA under biofilm-enhancing conditions. Supernatants were isolated from cultures (at a similar OD_600_) of WT, Δ*cprS*, Δ*flhA*, or Δ*cprS* Δ*flhA* grown in either MH alone or MH/DOC. Equal volumes were used as templates for qPCR. DNA amounts are normalized to WT in MH alone. Error bars represent the mean of three separate cultures. *p<0.0001; **p = 0.0015; NS p = 0.42, unpaired t-test. **B**) Lysis, independent of flagella, occurs under conditions that promote biofilms. Cell-free supernatants were isolated as in above. Both total cellular protein (‘Bacteria’) and supernatants (‘Media’) were analyzed by Western blotting with an antibody specific for the cytoplasmic response regulator CosR.

To confirm microscopy observations that suggested more eDNA was present under biofilm-enhanced conditions, the relative DNA concentration of culture supernatants was measured (**Fig. 2A**). Strains were grown overnight, and supernatants were harvested and subjected to qPCR with primers specific for the chromosomal *cprR* gene. For supernatants of biofilm-enhanced bacteria (Δ*cprS* and WT in MH/DOC), an approximately 3-4-fold increase in DNA was measured compared to WT in MH only (**Fig. 2A**; left three bars). Supernatants harvested from Δ*cprS* contained 3.2-fold more DNA than WT (p<0.0001). Similarly, when WT was grown in MH/DOC, bacteria released 3.8-fold higher levels of DNA than in MH alone (p = 0.0015). These values were consistent with microscopy observations (**Fig. 1**). We also did not observe an effect of flagellar mutation on the amount of DNA in the media (see below). This suggests that eDNA could be a quantitative marker for *C. jejuni* biofilm formation.

Microscopy suggested the mechanism underlying appearance of eDNA may be lytic, as can be the case in other bacteria [9], [37]. *C. jejuni* often displays a 2-log reduction in colony-forming units following log phase. This is enhanced in the Δ*cprS* mutant and was previously hypothesized to occur as a result of lysis [19]. The Δ*cprS* mutant also displays increased protein species in the media compared to WT during routine culture [19]. We hypothesized that lysis may underlie release of eDNA. Like Δ*cprS*, we observed increased protein in the media when WT was grown in the presence of sub-MIC levels of DOC (data not shown). We thus used Western blotting to detect the presence the cytosolic regulatory protein CosR [24] in culture supernatants (**Fig. 2B**). We did not observe differences in CosR expression in total cell protein samples. Supernatants from both Δ*cprS* and WT in MH/DOC contained significant levels of CosR compared to WT in MH only, which had undetectable levels. Detection of cytosolic proteins in supernatants strongly suggested lysis in Δ*cprS*, and WT in MH/DOC. However, the flagellar export apparatus has been reported to act as a type III secretion system-like machine in *C. jejuni*, secreting non-flagellar proteins into the medium and host cells. [38], [39]. Furthermore, DOC stimulates secretion of some flagellar-secreted proteins [40], and expression of FlaA is upregulated in Δ*cprS* [19]. Thus, we wanted to rule out flagella-dependent secretion of CosR under biofilm and DNA release-promoting conditions. As such, WT and Δ*cprS* strains harboring a deletion of the flagellar export apparatus gene *flhA* [39] were also included in Western blot analyses (**Fig. 2B** **;** lanes denoted Δ*flhA*). Importantly, we observed CosR in supernatants when Δ*flhA* was introduced into either Δ*cprS* or WT in DOC. Together, these observations suggested that a lytic mechanism, occurring independently of flagella-mediated export, underlies the appearance of both eDNA (**Fig. 2A**; right three bars) and protein (**Fig. 2B**) in supernatants of biofilm-enhanced *C. jejuni*.

### Addition of exogenous DNA enhances biofilms; removal of eDNA inhibits biofilm formation

**Figure 3:**
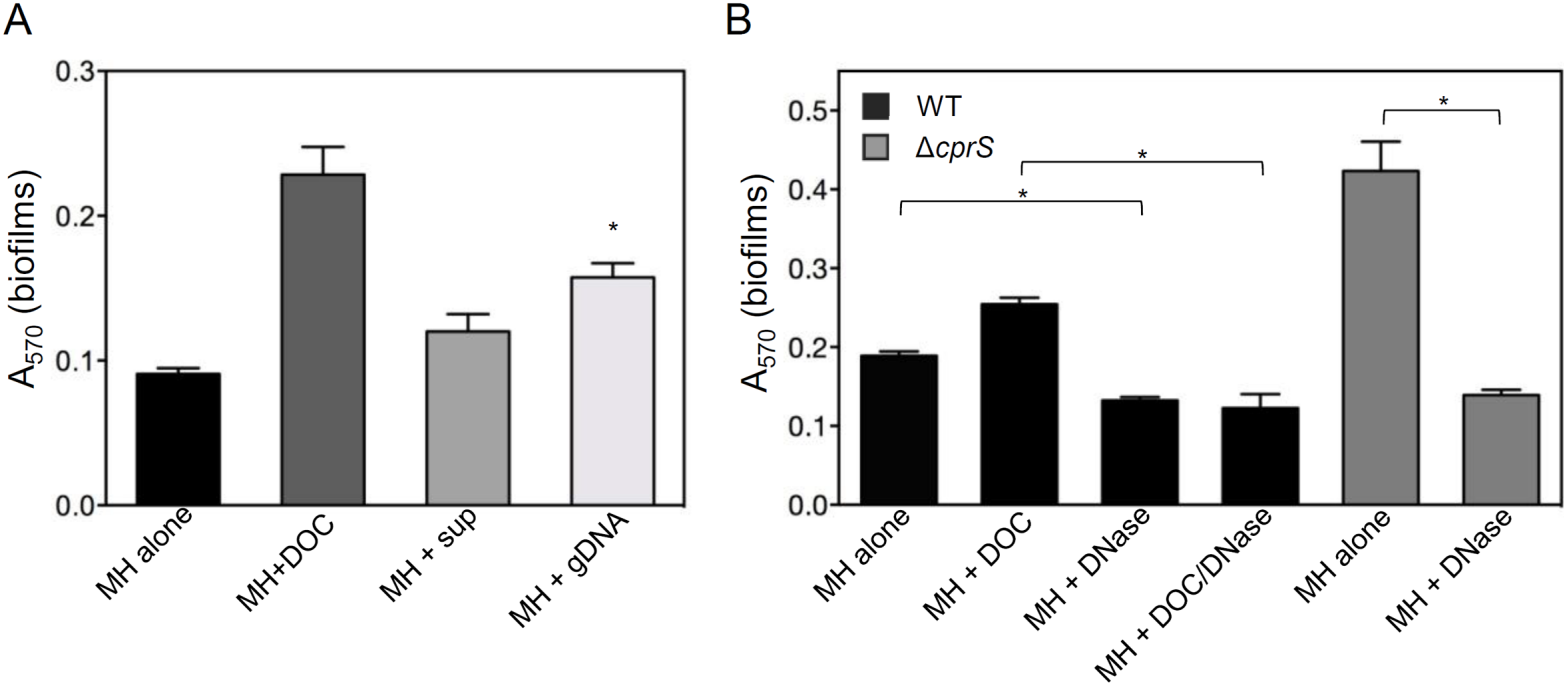
Cell-free supernatants and exogenous DNA promote biofilms and DNA is necessary for biofilm formation. **A**) Exogenous DNA enhances biofilms. Culture supernatants, concentrated for >3 kDa size components, or gDNA isolated from WT *C. jejuni* grown for 24 h on MH plates (500 ng) were included in fresh MH broth. Tubes were then inoculated with WT, and following 2 days growth, biofilms were quantified with crystal violet. *p = 0.08; **p = 0.003 (vs. MH alone). **B**) Biofilm formation is inhibited by DNase I. Biofilms (WT/black bars or Δ*cprS*/grey bars) were grown in either MH alone, MH/DOC, (0.05%) MH/DNase (90 U mL^−1^), or MH/DOC/DNase, followed by CV staining after 2 days growth. Error bars represent the mean of three biological replicates. *p<0.005 vs. counterpart without DNase.

Although DNA release correlated with biofilm phenotype, it was still unclear whether eDNA contributed mechanistically to biofilm formation. Pre-formed *C. jejuni* biofilms can be disrupted with DNase I, suggesting that like for other bacteria, eDNA plays a functional role in these structures [19]. To determine if eDNA affects the *C. jejuni* biofilm formation process, we used a crystal violet assay standard to our laboratory [19], [20], [22], [23], [34], [35] to test the effect of adding exogenous *C. jejuni* genomic DNA to standing cultures (**Fig. 3A**). The effect of adding cell-free culture supernatants, which contain eDNA, was also examined. We consistently observed that culture supernatants modestly enhanced biofilm formation (MH/sup), although this difference did not reach statistical significance (p = 0.08). The spent media (1/20 volume 10-fold concentrated supernatants was added) also appeared to partially inhibit growth. We thus determined the effect of added DNA alone. Purified *C. jejuni* genomic DNA was added to WT biofilm cultures (MH/gDNA), and these cultures showed a significant increase in biofilm formation compared to those grown in broth alone (p = 0.003). We also performed the complementary experiment and asked whether endogenous DNA was required for biofilm formation (**Fig. 3B**). Biofilm cultures of WT, Δ*cprS*, and WT in MH/DOC were grown in the presence or absence of DNase I. Biofilm formation by WT in DNase (MH/DNase) was reduced compared to that of WT in MH alone (p = 0.0013). Furthermore, when DNase was included in biofilm-enhanced cultures (Δ*cprS*; WT in MH/DOC), they formed significantly less biofilm than their counterparts grown without DNase (p = 0.0017, p = 0.0025, respectively. Collectively, these experiments suggested that eDNA release during biofilm formation was not simply a consequence of, but required for, biofilm formation.

### DNA is required for maturation of the *C. jejuni* biofilm

**Figure 4:**
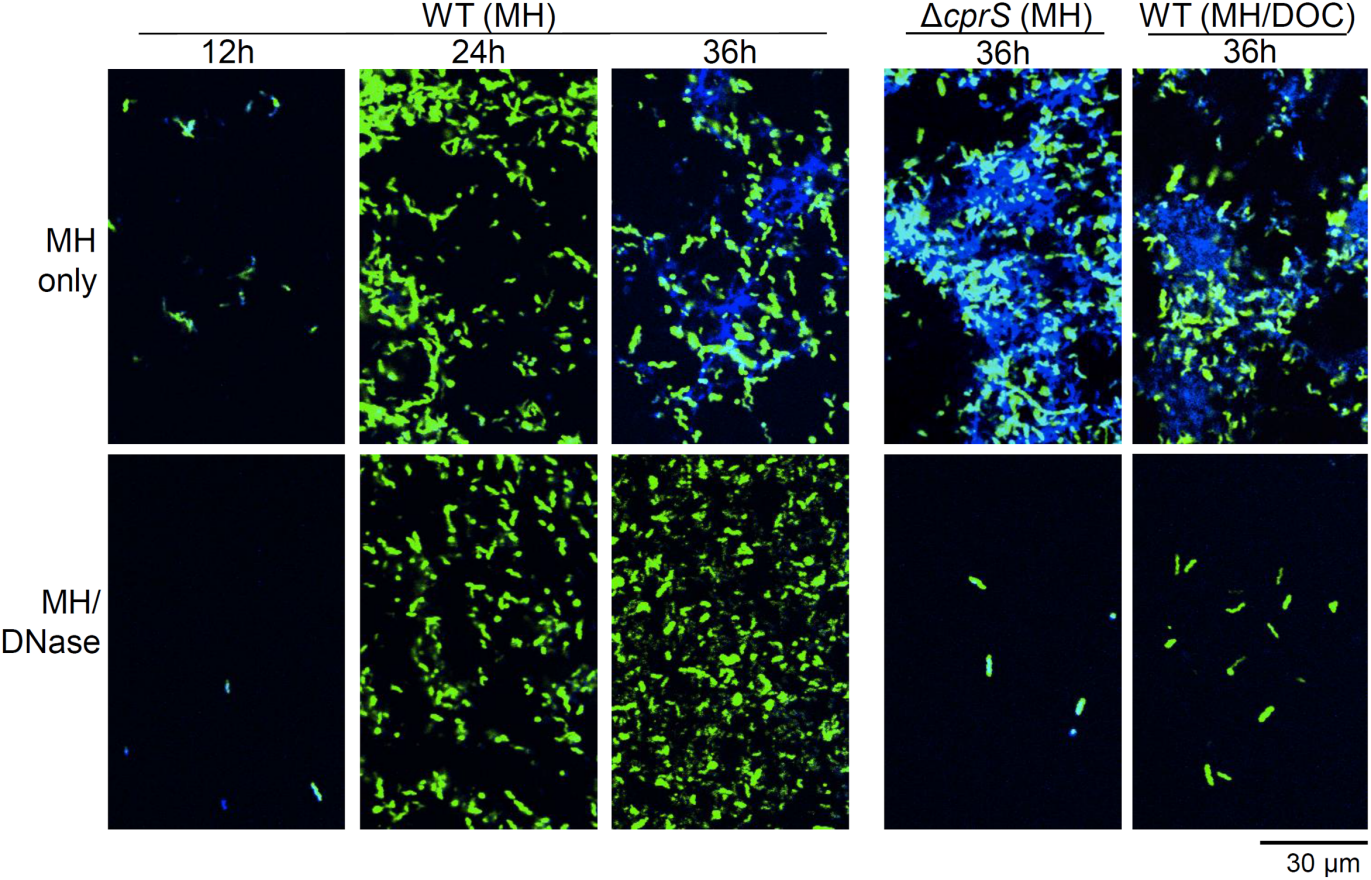
DNase arrests biofilms following adherence. Biofilms of WT, Δ*cprS*, or WT in MH/DOC (0.05%) were grown on coverslips in the presence (top panels) or absence (bottom panels) of DNase (90 U mL^−1^). After the indicated times, biofilms were fixed, stained with DAPI, and visualized by confocal microscopy. Green: GFP-expressing bacteria; Blue: DAPI-stained DNA.

The relatively low sensitivity of the crystal violet assay prevented us from determining if DNase completely inhibited biofilm formation, or if it arrested it at a very early stage of development. To determine at which stage biofilms were arrested by DNase, and to confirm that eDNA was in fact being degraded by the addition of DNase, we used confocal microscopy to observe biofilm formation in the presence and absence of the enzyme (**Fig. 4**). For WT (left panels, 12 h, 24 h, and 36 h), DAPI-stained DNA surrounding cells observed in MH alone was not observed when DNase was included, suggesting that the enzyme was sufficiently active. However, a few cells that were not expressing green GFP were blue, as the DAPI was presumably able to enter the cells and stain chromosomal DNA, but DNase was too large to enter. Unlike what was suggested by the low-resolution crystal violet assay above (**Fig. 3B**), confocal microscopy showed that DNase did not completely eliminate biofilm formation. Closer observation suggested that DNase arrested WT biofilms following adherence. Cultures with DNase included were still adhered to the coverslip (bottom three panels), but they remained in a monolayer and did not progress to more elaborate structures observed in MH only in the same time period (top three panels). Thus, the crystal violet assay, used extensively to measure biofilm formation in multiple bacterial species, may not be sensitive enough to detect the initial adherence step in *C. jejuni*.

Data in Fig. 3 showed that, by the crystal violet assay, biofilm-enhanced cultures (Δ*cprS*, WT in MH/DOC; right panels) displayed a similar inhibition of biofilm formation upon inclusion of DNase as WT under routine conditions. By confocal microscopy, however, addition of DNase not only inhibited biofilm formation in Δ*cprS* and WT (MH/DOC), but very few adherent bacteria were observed. Based on this observation, together with poor growth of Δ*cprS* Δ*flhA* during the above experiment (**Fig. 2A**), we hypothesized the biofilm may provide *C. jejuni* with fitness that is especially required by Δ*cprS* and WT in MH/DOC. This was addressed in subsequent experiments (below). Nonetheless, our observations are consistent with removal of DNA arresting biofilm formation following attachment, and having little effect on adherence of WT.

### Flagella are necessary for biofilm formation by both WT and biofilm-enhanced *C. jejuni*

**Figure 5:**
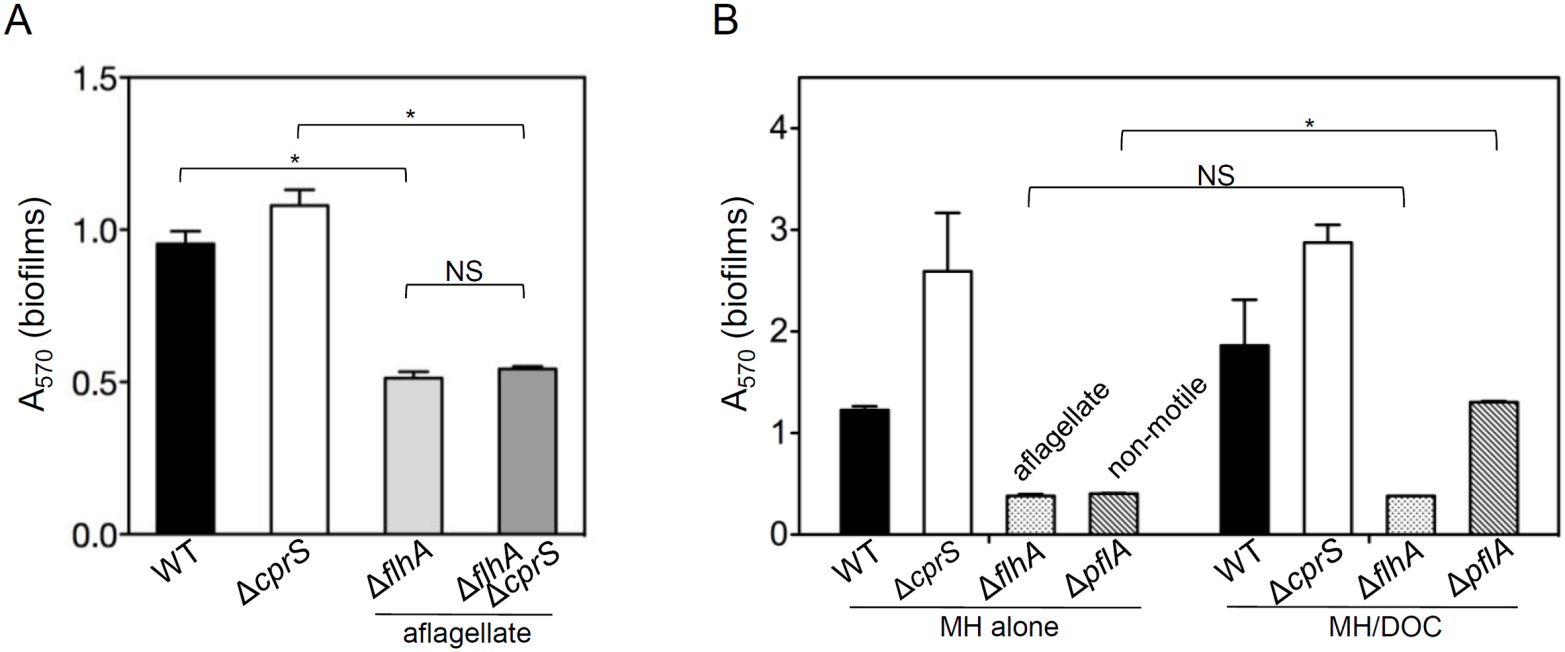
The flagellum, but not motility, is absolutely required for biofilm formation. **A**) Aflagellate mutants are defective for biofilm formation in WT and Δ*cprS* backgrounds. *p<0.0001; NS p>0.1 **B**) Only non-flagellate bacteria remain completely defective in biofilm-promoting media. *p<0.0001; NS, p = 1. Indicated strains were grown in static culture for 2 days in either MH broth alone or MH/DOC, followed by staining and quantification with crystal violet.

As we observed release of DNA at later time points, following adherence, we next sought to identify a factor required for the initial attachment step. Mutations that cause an aflagellate phenotype have consistently been reported to cause defective biofilm formation in *C. jejuni* [17], [25], [26]. As mutation of flagella did not affect lysis or release of eDNA (**Fig. 2**), we hypothesized that flagella may be required in a step prior to the stage where eDNA is relevant – specifically, adherence. To test this, biofilm formation by flagellar mutants was assessed (**Fig. 5A**). Deletion of *flhA* caused severely defective biofilm formation. When introduced into a Δ*cprS* background, the Δ*flhA* mutation also resulted in defective biofilm formation, suggesting flagella are epistatic to Δ*cprS*. Thus, we confirmed the biofilm-defective phenotype upon loss of flagella, and also showed that flagella were absolutely required for biofilm formation, even under conditions that can enhance biofilms.

### The flagellar filament is required for attachment; motility aids kinetics of biofilm formation

In addition to motility, in the absence of structures such as pili, *C. jejuni* flagella also appear to mediate adhesion [28]. We therefore sought to determine if motility or the flagellar structure was required. As a Δ*flhA* mutant is aflagellate [26] a *pflA* mutant, that expresses paralyzed flagella [41], was included in our analyses. In MH only (**Fig. 5B**, left), Δ*flhA* and Δ*pflA* were both markedly defective for biofilm formation compared to WT and Δ*cprS*. However, in MH/DOC (**Fig. 5B**, right), these strains displayed distinct behaviour. While the Δ*flhA* mutant remained biofilm-defective in MH/DOC, Δ*pflA* was not as defective, displaying a significant 3-fold increase in biofilm formation in MH/DOC compared to MH only (p<0.0001). The “+DOC” observations suggested that loss of motility could be partially rescued in conditions that promote biofilm formation in *C. jejuni*, but only in the presence of the flagellin adhesin.

**Figure 6:**
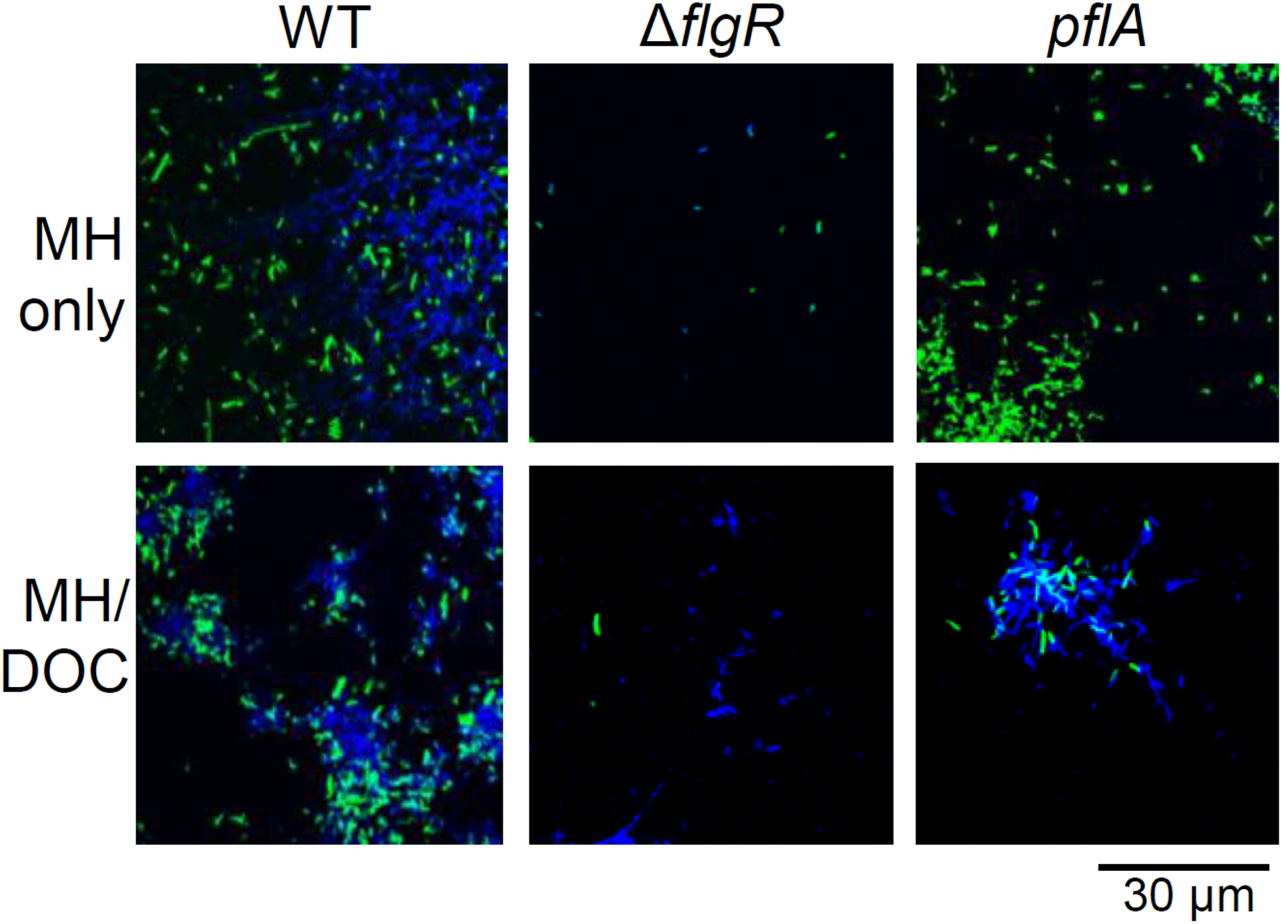
Aflagellate bacteria are defective for adherence; kinetics of biofilm formation is delayed in bacteria expressing paralyzed flagella. Biofilms of WT, Δ*flgR* (aflagellate), and *pflA* (paralyzed flagella) were grown on coverslips for 36 h, fixed, stained with DAPI, and visualized by confocal microscopy. Green: GFP-expressing bacteria; Blue: DAPI-stained DNA.

Microscopy was used to determine at which stages flagella and motility might contribute to biofilm formation. An aflagellate Δ*flgR* mutant (Kan^R^), that is also biofilm defective (data not shown), was used in place of Δ*flhA* to allow introduction of GFP on a Cm^R^ plasmid for microscopy. In MH only, the aflagellate Δ*flgR* mutant adhered poorly to coverslips compared to WT, with very few green bacteria observed attached to the slide (**Fig. 6**, top left and top middle panels, respectively). In contrast, more adherent Δ*pflA* bacteria were seen than for Δ*flgR* (top right panel). However, in contrast to WT, Δ*pflA* still appeared defective and/or delayed for both adherence and biofilm formation. Fewer adhered bacteria and little DNA were observed compared to WT at the same time point. Although very few bacteria were observed for Δ*flgR*, DNA was still observed attached to Δ*flgR*-incubated slides, confirming the above observations (**Fig. 2A**) that DNA release was not abolished by loss of flagella. Consistent with crystal violet results, inclusion of DOC in MH broth appeared to allow the flagellate, but non-motile Δ*pflA* mutant (bottom right panel) to form better biofilms, although still not to the levels of WT. Significantly more DNA were observed surrounding Δ*pflA* in MH/DOC compared to MH only. Thus, the defect observed in non-motile bacteria can be partially rescued by stimulating biofilm formation with conditions that enhance lysis and eDNA release, such in MH/DOC. Thus, this suggests that *C. jejuni* absolutely requires the flagellar structure to initiate biofilm formation, presumably to mediate adherence, and that eDNA is not sufficient to mediate adherence. While motility is dispensable under certain conditions, we conclude that it aids the kinetics of biofilm formation.

### Biofilms contribute to fitness under adverse conditions *in vitro*

In experiments described above (**Fig. 4**), WT/DOC or Δ*cprS* biofilm cultures incubated with DNase displayed very few bacteria adhering to the coverslip. Closer inspection of biofilm cultures suggested that while each strain was able to grow in the sub-MIC levels of DOC, there appeared to be a decrease in total biomass produced by cultures that were both biofilm-inhibited and experiencing ‘stress,’ compared to those that were only biofilm-inhibited (for example, WT in DNase vs. WT in both DOC and DNase). There have also been reports in the literature of *C. jejuni* flagellar mutant strains (such as Δ*rpoN* and Δ*fliA*) that are likely biofilm-impaired that display growth and stress tolerance defects in standing culture [42], [43]. Together, this suggested to us that *C. jejuni* requires biofilm formation to tolerate adverse conditions *in vitro*.

To test this, we determined if strains showed decreased fitness in the presence of a pathogenesis-related condition that normally did not markedly affect growth (sub-MIC DOC) if they were not able to form a biofilm, as measured above (**Figs. 3** **–** **6**). As a measure of fitness, we used the total biomass that was reached for each strain/condition during standing culture, similar conditions to our biofilm assay.

**Figure 7:**
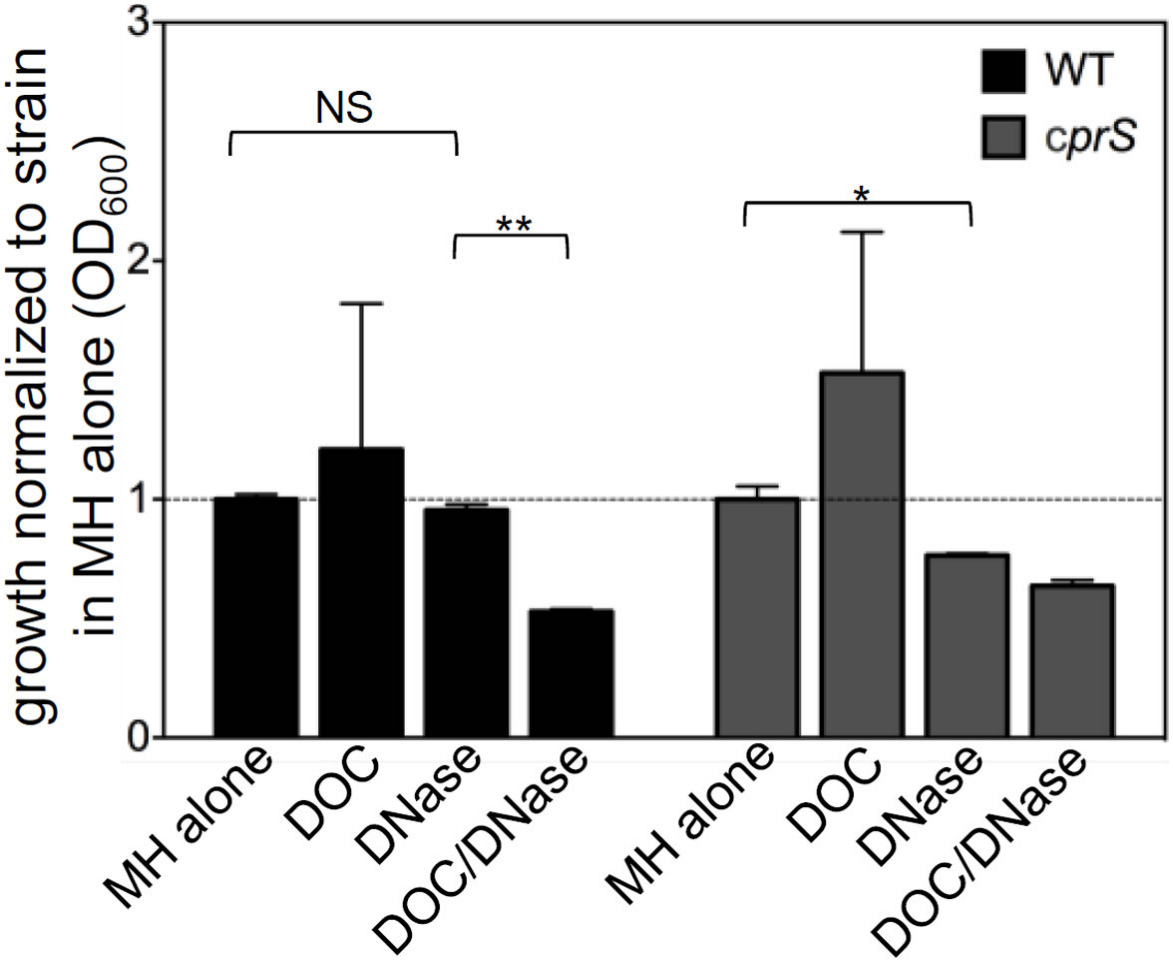
Biofilm formation confers stress tolerance *in vitro*. Standing cultures of the indicated strains (black bars, WT background; grey bars, Δ*cprS* background) were grown in MH broth with the indicated additions (labels below). Biofilm formation was impaired by addition of DNase (90 U mL^−1^). Sub-MIC levels of DOC were included where indicated. Total OD_600_ of three independent cultures, following 2 days growth and resuspension by vortexing was measured. Cultures were normalized to the strain background (WT or Δ*cprS*) in MH alone. Error bars represent the mean of three biological replicates. NS: not significant **p<0.0001 *p = 0.0018.

We first sought to determine the requirement of flagella-mediated adhesion for adaptation to DOC. However, aflagellate strains reached much lower total biomasses than flagellate strains, which made comparisons difficult (data not shown). We thus focused on the requirement for biofilm maturation (ie, the effect of DNase, which arrests biofilm formation following adherence). We noted no difference in growth (OD_600_ of resuspended cultures) for WT in MH or MH/DNase (**Fig. 7**, black bars), suggesting that DNase did not appreciably affect fitness of WT under routine conditions. In contrast, when DOC was included along with DNase, we saw a significant decrease in final biomass (p<0.0001) reached by WT. Sub-MIC levels of DOC increased total resuspended culture density, together with reduced density of flagellar mutants (see above) suggested that biofilm formation allows *C. jejuni* to reach higher bacterial loads.

We also included Δ*cprS*, which like WT in DOC is presumably experiencing stress due to absence of CprRS signaling [19], as a comparison (**Fig. 7**, grey bars). Like WT, Δ*cprS* reached a higher resuspended culture density in the presence of DOC, compared to MH alone. However, in contrast to WT, Δ*cprS* was significantly affected by inclusion of DNase in the culture media, even in the absence of DOC (p = 0.0018). As Δ*cprS* shows numerous *in vitro* stress-related phenotypes, including reduced tolerance of osmotic and oxidative stress [19], this suggests that its enhanced biofilm phenotype may be a compensatory stress response. Together, our *in vitro* observations suggest that *C. jejuni* requires biofilm formation for fitness in the face of challenging conditions.

### Conditions that increase DNA release and biofilms also promote recombination

**Figure 8:**
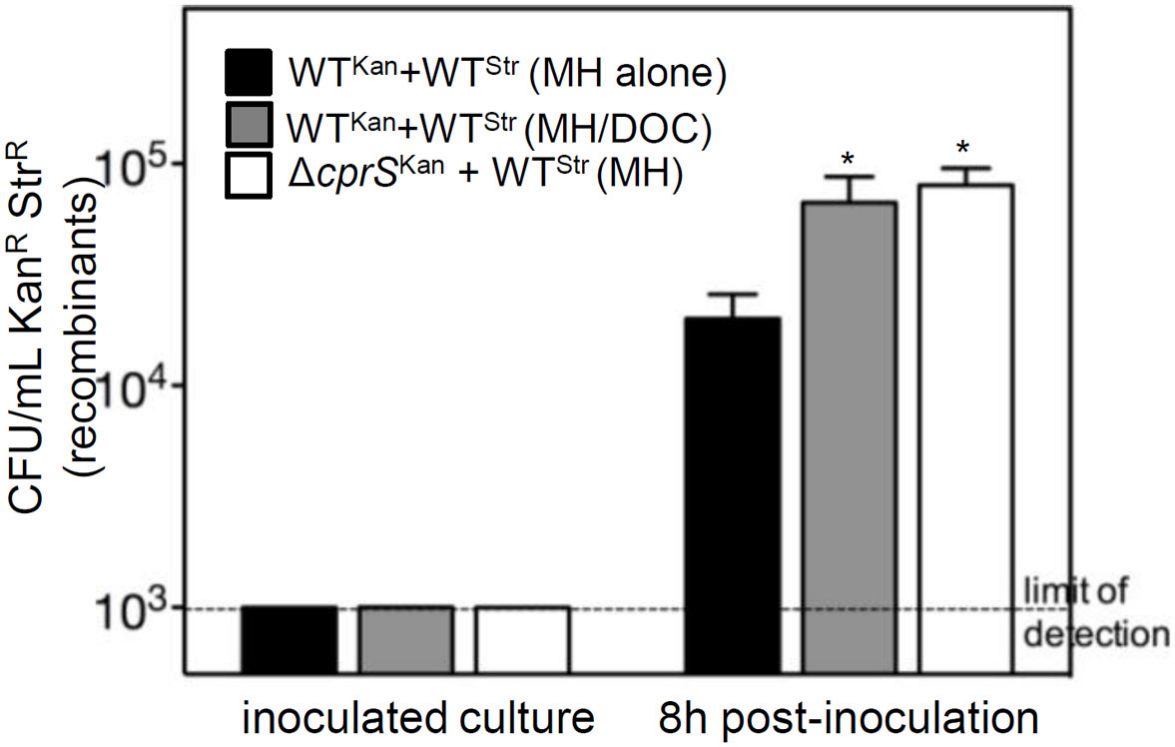
Conditions that promote DNA release and biofilms also increase genetic exchange and UV tolerance. Genetic exchange. WT bacteria, marked with Str^R^, were grown in mixed culture (1∶1) with either an isogenic WT strain marked with Kan^R^ or the Δ*cprS* mutant marked with Kan^R^. Cultures were grown in either MH broth alone or MH/DOC. Cells were removed at indicated time points and CFUs were determined on the appropriate antibiotics. Error bars represent the mean of three biological replicates. *p<0.1 vs. WT+WT (MH).

As eDNA was increased under conditions that promoted biofilm formation (**Fig. 1C**), we asked whether increased extracellular DNA could also increase horizontal gene transfer. Genetic exchange was measured under two conditions that promote biofilm formation and eDNA release: mutation of *cprS*, and growth in MH/DOC. Strains marked with antibiotic resistance (Δ*cprS*, Kan^R^, WT, Str^R^ or Kan^R^) on the chromosome were grown in mixed culture. When WT (Str^R^) was grown with WT (Kan^R^), the appearance of doubly resistant colonies, not present upon inoculation of the cultures, was observed (**Fig. 8**). When the same mixed cultures were grown in MH/DOC, appearance of more of these doubly-resistant clones was observed compared to cultures in MH alone (p = 0.09). Moreover, when WT (Str^R^) was co-cultured with Δ*cprS* (Kan^R^), we also recovered significantly more (p = 0.02) colonies on plates containing both Kan and Str compared to those from cultures of the two WT strains.

## Discussion

Previous work had not identified dedicated virulence factors or specific stress response proteins that sufficiently explain why *C. jejuni* is such a successful zoonotic pathogen, surviving and thriving in numerous environments during transmission and pathogenesis. In this work, analysis of strains enhanced for biofilm formation (Δ*cprS*; WT in MH/DOC) identified stages and molecular factors involved in *C. jejuni* biofilm formation, a phenomenon that may explain the resilience of *C. jejuni* outside of the laboratory. Two specific phenomena that appear to be related to *C. jejuni* biofilm formation, at least *in vitro*, were determined: flagella and eDNA. Flagella appear to be necessary for initiation of biofilm formation on a surface by mediating adhesion. Furthermore, motility provided by flagella also aided kinetics of biofilm formation. We also observed a lytic phenomenon that correlates with biofilm formation and appears to be responsible for release of eDNA. We also found that eDNA was then required for maturation from microcolonies into a three-dimensional biofilm. Finally, we observed that inhibition of biofilm formation lead to reduced fitness in the presence of DOC, a pathogenesis-related stress that also appears to trigger *C. jejuni* biofilm formation.

The process of biofilm formation in *C. jejuni*, like other bacteria, appears to proceed in discrete steps, starting with adhesion. We propose that flagella are required for adhesion, as aflagellate mutants were not observed to adhere to coverslips, even under conditions that normally enhance biofilm formation of WT (such as with DOC) (**Fig. 6**). This is consistent with two previous studies have noted that bacteria adhere to *ex vivo* tissue samples by flagella in microcolony-like structures [27], [29]. Analysis of biofilm formation on abiotic surfaces also found microcolonies formed on glass coverslips with flagella forming bridges between organisms [30]. Moreover, autoagglutination, which is thought to be dependent on flagella and biofilm formation, also seem to be correlated in *C. jejuni* [18], [44].

A central role for flagella in biofilm formation is also supported by previously reported expression data. Motility peaks during late log phase [45], and Class II and III flagellar genes exhibit sustained or increasing expression through stationary phase, suggesting components of the flagellum may be necessary for this transition. Biofilm cells often exhibit characteristics of stationary phase cells and share similar expression profiles [46], and *C. jejuni* biofilm cells also display higher expression of flagellar genes compared to stationary phase cells grown planktonically [26]. Finally, proteomic and microarray expression analysis of the Δ*cprS* hyper-biofilm mutant [[19]; S.L. Svensson and E.C. Gaynor, in preparation] also suggest expression of flagellar genes is increased in this strain.

It was initially unclear whether motility or the flagellum itself was required for adhesion. Further mutant analyses using a paralyzed flagellum mutant suggested that while motility might aid the kinetics of biofilm formation, it was not absolutely required. In contrast, the flagellum structure itself was. Our observations are consistent with behaviour of other mutants with a variety flagellar morphologies and motilities [17], [26]. Biofilm formation is consistently severely defective in aflagellate mutants (such as Δ*flhA*), but delayed in strains such as Δ*flaA,* Δ*flaB,* Δ*fliA*, and Δ*flaC* [26]. These strains express either normal or morphologically aberrant flagella and have reduced (∼20% of WT) or absent motility [26], [47]. Interestingly, a Δ*flaG* mutant, which expresses long flagella but retains full motility, is completely defective for biofilm formation, even upon extended incubation [26]. This suggests that motility alone may be insufficient for biofilm formation, and that aspects of the flagellar structure itself are critical for biofilm formation.

We observed release of eDNA following adherence and found that it is required for further maturation of the biofilm. DNase did not appear to affect the initial adherence step. Consistent with this, *C. jejuni* has been proposed to use both flagellum-dependent and -independent mechanisms of biofilm formation [18]. In other bacteria, adherence is often followed by biogenesis or release of polymeric matrix components that encase the mature biofilm. Surface carbohydrates are common components of biofilm matrices, and the *C. jejuni* surface is highly glycosylated. It is therefore puzzling that a specific carbohydrate component of the *C. jejuni* matrix has yet to be identified. We previously noted that DNA surrounds *C. jejuni* biofilms, especially in Δ*cprS* and in WT bacteria under conditions favouring biofilm formation (MH/DOC), and treatment of pre-formed biofilms with DNase also disrupted them [19]. An extracellular material that binds Ruthenium Red [30], a dye that stains carbohydrate matrices, but also binds double-helical DNA, was previously observed [48]. The Δ*cprS* mutant carries no gross defects in surface polysaccharides [19]. We have now measured a 2-3-fold increase in eDNA for Δ*cprS* compared to WT after the same incubation period.

We have also shown that exogenous, purified *C. jejuni* gDNA enhances biofilms, and inclusion of DNase in standing cultures inhibits biofilm formation (**Fig. 3**). Thus, it appears that eDNA does in fact play an important role in *C. jejuni* biofilm formation, and does not simply correlate with the transition to a sessile lifestyle. Consistent with this, the presence and important role of eDNA in biofilms is now well-appreciated in many species. It is interesting to note that deletion of *dps*, encoding a nucleoid-binding protein, reduces biofilm formation by 50% [49]. Unlike addition of *C. jejuni* gDNA, highly purified salmon DNA does not enhance biofilm formation (data not shown). It is possible that chromatin-like material, possibly containing proteins like Dps, may serve as an enucleating factor for biofilm maturation. We cannot rule out a potential contribution of other proteins released during lysis in *C. jejuni* biofilms. The enhanced biofilm phenotype of many loss-of-function mutants in surface carbohydrate loci of *C. jejuni* is intriguing and suggests that biofilm formation in this organism does not require a specific carbohydrate matrix component. Expression of a particular surface carbohydrate may instead be negatively correlated with biofilm formation, such as glycosylation of flagella or the major outer membrane protein [44], [50], which would change surface hydrophobicity. Alternatively, DNA may fulfill the role played by exopolysaccharides in other bacteria, or a carbohydrate component, which may not be absolutely required under laboratory conditions, could be provided by a neighbouring organism in a multi-species biofilm in nature.

The source of the eDNA is unknown; however, we noted co-occurrence of increased eDNA with cytosolic proteins in culture supernatant (**Fig. 1**). An increase in many of protein species was previously noted in supernatants of Δ*cprS* [19]. The Δ*cprS* mutant displays a more marked loss of culturability following log phase compared to WT [19]. *C. jejuni* is thought to convert to a coccoid viable but non-culturable state; however, Δ*cprS* morphology is not consistent with an accelerated progression to this form [19]. Taken together, this implicates a lytic process. It is unknown whether the released DNA is chromosomal, consistent with lysis, or shows any enrichment for particular sequences. Furthermore, while DNA uptake appears to be mediated by a Type II secretion system, a putative DNA secretion apparatus has not been identified in *C. jejuni*. The pVIR plasmid carried by some strains, including the robust biofilm former 81–176, encodes a putative Type IV secretion system that could possibly mediate this, as in *Neisseria gonorrhoeae* [51]. However, mutation of *virB11*, encoding an essential component of this secretion system, does not affect biofilm formation in strain 81–176 (S.L. Svensson and E.C. Gaynor, unpublished observations). In the related gastric pathogen *H. pylori*, eDNA has also been identified as a component of the biofilm matrix [52]. DNA fingerprinting suggested a marked difference between eDNA and intracellular DNA, suggesting that a non-specific lytic mechanism does not release of DNA in this pathogen. However, DNase does not affect biofilm formation by *H. pylori*, and thus, it was concluded that the main function of eDNA in this bacterium was to contribute to genetic variation.

Our observations do not allow us to propose whether such a lytic mechanism is passive or autolytic. A connection between autolysis and biofilm formation exists in other bacteria. In *P. aeruginosa*, autolysis appears to contribute to dispersal of organisms from the biofilm, whereas in other bacteria such as *Enterococcus faecalis*, *Staphylococcus aureus*, and *N. meningitidis*, it appears to be involved in both eDNA release and biofilm development [8], [9], [53], [54]. Lytic transglycosylases in *Salmonella* Typhimurium also link cell wall turnover to biofilm formation [55]. Unfortunately, we did not observe any accessory autolysins in the *C. jejuni* that may provide support for a lytic mechanism. Instead, ‘housekeeping’ peptidoglycan modification enzymes may be involved. Such enzymes are only now being identified and characterized in *C. jejuni* [34], [35]. While a regulated autolysis program has not yet been described in *C. jejuni*, a decrease in CFUs (approximately 2 logs) is often observed in WT cultures after exponential phase of growth.

Biofilm formation by *C. jejuni* appears to be triggered under particular stress conditions. It was recently reported that aerobic conditions stimulate biofilm formation in *C. jejuni* [18], and bile upregulates the *flaA* flagellin promoter [56]. We previously reported that DOC, and other detergents, upregulate biofilm formation in *C. jejuni* [19]. Furthermore, there is a positive correlation between envelope perturbations, such as in Δ*kpsS*, Δ*waaF*, and Δ*spoT*, as well as WT grown in polymyxin B and ampicillin, and a tendency to form enhanced biofilms [[22]–[24] (S.L. Svensson and E.C. Gaynor, unpublished observations)]. A close relationship between envelope stress and biofilm formation exists in other pathogens. For example, it has been proposed that the Cpx-controlled envelope stress response of Gram-negative bacteria mediates biofilm formation [57]. Similar to *C. jejuni* in DOC, bile stimulates biofilm formation in *Vibrio cholerae* [58]. Interestingly, it has been shown that deletion of oxidative stress genes such as *ahpC* or catalase increases biofilm formation, where as overexpression of *ahpC* correlated with decreased biofilm formation [59]. Thus, biofilm formation may be a more general response to adverse conditions.

In support of observations that suggest biofilm formation is a stress response, we have also shown that inhibition of biofilm formation in *C. jejuni* increases the inhibitory effect of sub-MIC levels of DOC (**Fig. 7**). In general, we observed that bacteria that could not form a mature biofilm,either by genetic lesion of flagellar genes (data not shown) or enzymatic removal of eDNA, were less able to grow in standing culture with added DOC than those that could form a biofilm. Consistent with our observation, other work has shown that flagellar mutants (Δ*rpoN* and Δ*fliA*) exhibit growth differences and/or stress sensitivity in standing culture [38], [43]. Cultures that could form biofilms also reached higher total biomass than those growing solely planktonically, even in MH broth alone, suggesting that biofilms could presumably increase the burden of this pathogen in the environment. The mechanism by which biofilms conferred *C. jejuni* with increased stress tolerance in this work is currently unknown. In general, the contribution of biofilms to stress tolerance in other bacteria is thought to be multi-factorial, and may include altered metabolism, induction of stress response genes, changes in the cell envelope, decreased penetration of O_2_ or inhibitory compounds (such as DOC), or specific contributions of the properties of matrix components, such as eDNA. Nonetheless, it appears that the biofilm provides a niche well-suited to growth and/or survival of this pathogen, and conditions that promote biofilm formation may contribute to high bacterial loads in infection reservoirs. It also follows that antimicrobial agents may, to some extent, contribute to persistence of this pathogen by stimulating biofilm formation.

While our *in vitro* observations suggest that biofilm-residing *C. jejuni* are more stress tolerant, the role of biofilms *in vivo* has thus far been unclear. *C. jejuni* encounters numerous stresses in both commensal and susceptible hosts, and has been observed to form microcolonies on intestinal epithelial tissue *in vitro* [*29*]. Moreover, species of *Campylobacter* have been identified within biofilms in the upper gastrointestinal tract of patients with Barrett's esophagus [60], and *H. pylori* also forms biofilm-like structures in the gastric mucosa [61], [62]. Indirect evidence suggests biofilms may partially protect otherwise sensitive mutants of *C. jejuni*. A Δ*spoT* stringent response mutant forms enhanced biofilms[22], [63] and retains its capacity to colonize animal hosts, even though it displays specific *in vitro* stress-related defects (E. Gaynor, unpublished observations]. In addition, a Δ*ppk1* mutant, which also exhibits stress tolerance defects *in vitro*, displays a dose-dependent trend for both *in vitro* biofilm formation and chick colonization [20]. Collectively, this suggests that biofilms do confer stress-sensitive mutants with *in vivo* resilience.

In addition to tolerance of acute instances of stress, our observations suggest that the mechanism of *C. jejuni* biofilm formation support its high genetic diversity, which could contribute to longer-term adaptation to varying environmental conditions. *C. jejuni* exhibits phase variation of genes relating to its cell surface – genes that are critical to its interaction with the host environment - and this has been shown to occur during colonization of chicks [64]. Exchange of genetic markers has also been observed in chicks [65]. eDNA released under biofilm-promoting conditions has the potential to serve as a substrate for horizontal gene transfer, and we observed an increased rate of marker exchange under biofilm-promoting conditions. However, the reason for this may be multi-factorial, and it remains to be demonstrated whether processes such is DNA uptake and recombination may also be upregulated during biofilm-enhancing conditions. Autolysis can in fact be a trigger for natural transformation in other bacteria [66]. Importantly, we observed increased recombination in conditions that may be encountered during both colonization of commensal hosts and pathogenic infection of humans (i.e., presence of DOC). This suggests that such a mechanism may occur *in vivo*.

**Figure 9:**
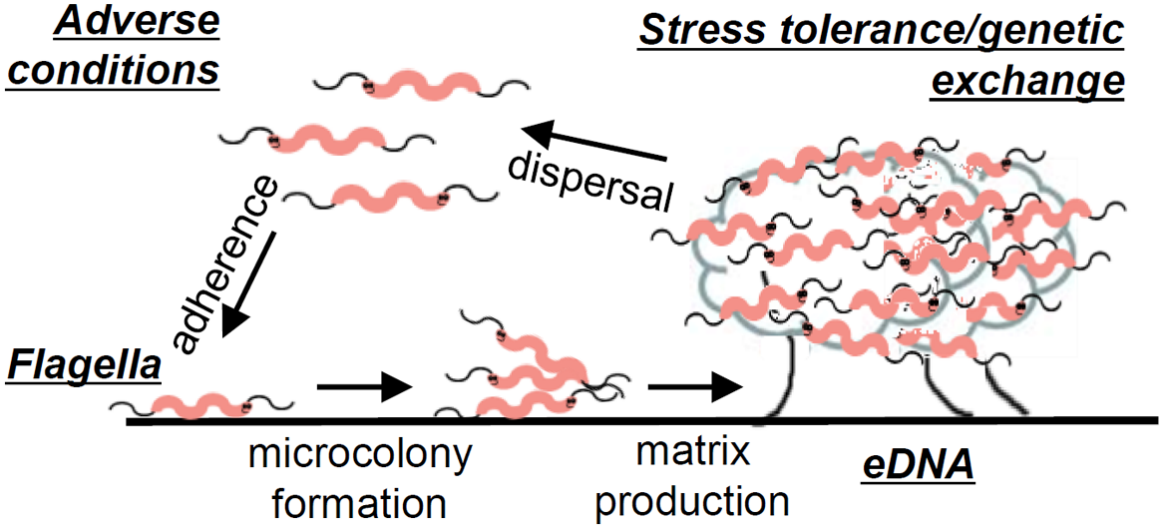
Model of *C. jejuni* biofilm formation. Evidence for the role of stress conditions, flagella and motility, eDNA release, and genetic exchange has been provided. Biofilm formation also appears to confer tolerance of specific stresses, such as those that may be encountered during pathogenesis.

In the absence of the large repertoire of survival factors expected for a zoonotic pathogen, global changes in physiology may underlie adaptation of *C. jejuni* to stressful environments. Phenotypes required for rapid growth are often expressed at the expense of stress tolerance [67]. Thus, some of the resilience of *C. jejuni* may not be observed planktonic broth culture, explaining the apparent fastidiousness of *C. jejuni* in the lab. In this work, we have extended understanding of the steps and molecular mechanisms of *C. jejuni* biofilm formation, a process that provides this pathogen with stress tolerance, providing a framework for future studies (**Fig. 9**). Further characterization of these mechanisms will contribute to our knowledge of how *C. jejuni* navigates environments encountered during pathogenesis.
